# Effects of *Alhagi maurorum* Medik polysaccharide derived from different regions on the intestinal immune functions of lambs

**DOI:** 10.3389/fphar.2024.1422461

**Published:** 2024-07-15

**Authors:** Zulikeyan Manafu, Ronglijiao Du, Xieraili Malajiang, Gulimire Abulikemu, Lijun Xue, Ayibike Bierdelieke, Yuan Xie, Dandan Liu, Zhanhai Mai, Qingyong Guo, Adelijiang Wusiman, Bin Li, Saifuding Abula

**Affiliations:** ^1^ College of Veterinary Medicine, Xinjiang Agricultural University, Urumqi, China; ^2^ Xinjiang Key Laboratory of New Drug Study and Creation for Herbivorous Animal, Xinjiang Agricultural University, Urumqi, China; ^3^ College of Grassland Science, Xinjiang Agricultural University, Urumqi, China

**Keywords:** Ficus carica L. polysaccharides, growth performance, intestinal immunity, intestinal microbiota, chickens

## Abstract

**Introduction:** Plant polysaccharide are widely studied as potential prebiotics because of their potential to protect and enhance the immunity of lambs.

**Methods:** In this study, the polysaccharide content of *Alhagi maurorum* Medik from Aksu (AK) and Shanshan (SS) at different cutting periods was determined, and the functions of *Alhagi maurorum* Medik polysaccharide were investigated to useas an immunomodulator.

**Results:** Our results indicated that the content of *Alhagi maurorum* Medik polysaccharide is the highest at the maturity stage, and the polysaccharide content of *Alhagi maurorum* Medik produced in Shanshan area is higher as compared to the Aksu area. The serum IgG, duodenum IgA, TNF-α, IL-4, IL-10 contents, jejunum IgA, TNF-α, IL-4, IL-17 contents, ileum IgA, IL-17 contents, duodenum villus height, crypt depth and jejunum crypt depth of lambs were significantly adjusted in the SS group as compared to CK control group and AK groups (*p* < 0.05). Furthemore, the sequencing results showed that SS polysaccharide promoted the release of large amounts of IgA and enhanced the immunal function of intestine by regulating the IgA production pathway and B-cell receptor signaling to activate B cells in the T-dependent pathway.

**Discussion:** Altogether, *Alhagi maurorum* Medik polysaccharide from SS group holds a promising potential to be used as a valuable immunopotentiator for optimizing the immune system of intestine in lambs.

## 1 Introduction

The intestine is a vital immune organ for animals to defend against pathogens (Ying et al., 2020; Cai et al., 2022b). The mucous of intestinal is an immune barrier which plays an important role in maintaining the diverse physiological functions and homeostasis within the intestine (Burgueño and Abreu, 2020; Wu et al., 2022), which consists of mechanical, biochemical and biological components (Beumer and Clevers, 2021). The biochemical barrier includes digestive juices, immunoglobulin A (sIgA) secreted by the cells of immune system and other substances that inhibit the adherence of pathogenic microorganisms (Garcia et al., 2018; Kumar et al., 2020). Among the biochemical barriers, IgA is a main defense antibody of the intestinal mucosal surface, which protects the delicate intestinal lining by forming a protective layer on the surface of the intestinal mucosa, neutralizing from potential pathogens (Li et al., 2020; Huus et al., 2021; Conrey et al., 2023). Intestinal IgA production is regulated by many factors, such as intestinal immune cells and cytokines. After that stimulation factor interacts with DCs, it induces the expression of BAFF and the April ligand, or MHCII. BAFF and April interact with TACI to induce IgA secretion on B1 cells, or/and MHCII act on CD4 T cells to transmit signals to B2 cells to induce IgA secretion, respectively (Huus et al., 2021).

Polysaccharide derived from natural plants have gained considerable attention in recent years as a key bio-active metabolite in traditional Chinese medicine. Their outstanding pharmacological effects made them valuable for various health applications (Xue et al., 2023). Plant-derived polysaccharide act as natural immune modulators by activating immune cells, interacting with the complement system, and releasing cytokines (Wu et al., 2019; Wang et al., 2023). These bio-active metabolites play an important role in supporting overall health and immune functions (Wu et al., 2019; Wang et al., 2023). Plant polysaccharide act as an environmentally-friendly feed additives, helping to reduce stress and improve the immune function in ruminants. Zhao et al., reported that supplementing the diet of calves with *Moringa oleifera* leaf polysaccharide significantly increased the villus height of calves, which promoted the intestinal IgA secretion (Zhao et al., 2023). Cai et al., reported that gavage of *Alhagi maurorum* Medik fruit polysaccharide significantly increased mouse villus length, decreased crypt depth, increased the proportion of IgA + cells and sIgA antibody in the intestine, and increased the TNF-α, INF-γ, IL-4, IL-17, and IL-10 contents and mRNA expression levels in the intestine (Cai et al., 2021b; Cai et al., 2022c). These polysaccharide boost the body’s defens against infection and disease by activating immune cells. Therefore, this study is planned for dietary supplementation with plant polysaccharide to improves disease resistance in ruminants. The plant-based polysaccharide can robust up the immune systems to defend the pathogens (Bai et al., 2023).

*Alhagi maurorum* Medik is mainly distributed in Central Asia and Northwest East Asia, and it is widely distributed in Northwest China, especially in Shanshan and Aksu in Xinjiang (Nabeela Ahmad et al., 2015; Yin et al., 2022). *Alhagi maurorum* Medik polysaccharide is rich in metabolites such as polysaccharide, alkaloids, flavonoids, anthraquinones and glycosides. *Alhagi maurorum* Medik has been widely used in folk medicine to treat mild diarrhea, abdominal pain, tongue ulcers and edema, and it has anti-inflammatory and diuretic effects (Marashdah et al., 2006; Marashdah and Al-Hazimi, 2010). *Alhagi maurorum* Medik thorn is used in veterinary medicine to treat gastrointestinal diseases in cattle, sheep, goats and camels (Khan, 2009). Previous studies have reported that *A. maurorum* Medik polysaccharide can stimulate the release of TNF-α in RAW264.7 macrophages and enhance IL-10 in immunosuppressed mice (Long et al., 2020). It is studied that *A. maurorum* Medik polysaccharide can improve the intestinal health and growth in mice and broilers by regulating the abundance of beneficial bacteria and reducing the number of harmful bacteria (Cai et al., 2022a). The intestinal immunity of mice and broilers was enhanced by the releasing of intestinal cytokines, height of facial features and depth of crypts, activation of intestinal immune cells, and increasing secretion of IgA in the intestine (Cai et al., 2022a). But currently there is limited research on the immune-enhancing effects of AP in different regions and the mechanism by which AP enhances intestinal immunity in lambs.

Therefore, in this study, AP was orally administered to lambs derived from the Shanshan (SS) and Aksu (AK) regions. The effects of SS and AK on the intestinal immune function of lambs were compared by the level of antibodies and cytokines secretion in the intestine. Finally, the effects of the polysaccharide on intestinal immune-related pathways and gene expression in lambs were analyzed by genome sequencing and Q-PCR was verified.

## 2 Materials and methods

### 2.1 Materials

Aksu and Shanshan are located in Xinjiang Province, which is rich in *A. maurorum* Medik resources (Table 1). The whole *A. maurorum* Medik grasses including stems, leaves and flowers were collected at the vegetative stage (May), flowering stage (August), maturity stage (September) and withered stage (October). The collected samples were dried in shady area for further use. All the *A. maurorum* Medik samples were morphological identified by Ma Shengjun (associate professor) from Xinjiang Agricultural University, and the mature stage *A. maurorum* Medik samples were used for further fingerprinting identification (S 1).

**TABLE 1:** *Alhagi maurorum* Medik sampling location information.

Sampling site	Longitude	Latitude	Altitude(m)
Sanhe Town, Awati County, Aksu City	80°13′ 43.79″	40°16′ 59.52″	1035.88
Dikan Town, Shanshan County, Turpan City	89°47′ 20.82″	42°35′ 43.11″	128.2

### 2.2 Preparation of *Alhagi maurorum* Medik polysaccharide

The preparation steps for *A. maurorum* Medik polysaccharide are as follows: the dried *A. maurorum* Medik sample is crushed, then put into distilled water with 12 times the weight of the sample and extracted by boiling 2 times. The extracted liquid is collected, filtered and concentrated, and anhydrous ethanol is added to the concentrate liquid until the ethanol concentration reaches 80%. Then the solution was kept at 4°C overnight, and the crude polysaccharide was obtained after precipitation and drying. Crude polysaccharide was obtained from Aksu District (AK) and from Shanshan District (SS). The phenol sulfuric acid method used to estimate the polysaccharide contents in *A. maurorum* Medik in different regions and at different cutting periods (Cai et al., 2022c).

### 2.3 Animal experiments

**FIGURE 1:**
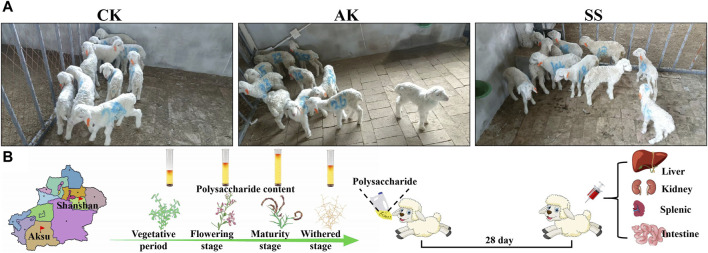
**(A)** Group of sheep lambs and **(B)** the process of animal testing.

The experiment was conducted at Puhui Township farm in Korla City. A total of thirty (*n* = 30) 5-day-old male Hu lambs with a body weight ranging from 2.6 to 2.9 kg were randomly divided into 3 groups, having 10 sheep lambs in each group (Figure 1A). In the control group (CK, administration of 20 mL of normal saline daily), gavage *A. maurorum* Medik polysaccharide produced in the Aksu group (AK, 20 mL of 1.5 g/kg AK aqueous solution daily), and gavage *A. maurorum* Medik polysaccharide produced in the Shanshan group (SS, 20 mL of 1.5 g/kg SS aqueous solution daily). The experiment duration was for 28 days, and on day 28, five lambs were randomly selected from each group. After taking the blood from vein, the lamb was euthanized by neck bleeding, and the following experiments were performed (Figure 1B). Animal experiments were performed in accordance with the approval of the experimental Animal Welfare Ethics Committee of Xinjiang Agricultural University (approval number: 2022016).

### 2.4 Detection of the sheep lamb organ index

On the 28th day of the experiment, the lamb’s liver, kidney, and spleen were collected and weighed to calculate the organ index.

$$\text{Organ index}=\text{organ mass}\left(g\right)/\text{lamb weight }\left(\text{kg}\right).$$

### 2.5 Detection of total IgG, total IgA and cytokines by ELISA

For quantification of total IgG and serum cytokines, 1 mL of whole blood collected from lambs was put at the room-temperature for an hour, and then serum was separated by centrifugation (2000 rpm, 15 min). To measure total intestinal sIgA and intestinal cytokines, a 1-cm section of the jejunum was obtained and mixed with 2 mL of PBS. The resulting supernatant was collected after centrifugation (3,000 rpm, 15 min). Serum lgG, serum IL-4, IL-10, IL-17, TNF-α, and INF-γ content, total jejunum IgA, jejunum IL-4, IL-10, IL-17, TNF-α, and INF-γ contents were measured using ELISA kits as per manufactures instruction (FANKEW, Shanghai Kexing Trading Co., Ltd, China).

### 2.6 Hematoxylin and eosin (H&E) staining detection

On day 28, the duodenum, jejunum, and spleen were immersed in a 4% paraformaldehyde fixative for fixation purposes. Then, the tissues were undergoing histological processing to obtain stained sections using a Nikon digital camera under a standard optical microscope at ×40 and ×200 magnifications, respectively. The villous height and intestinal crypt depth were measured using the ImageJ analysis system (NIH, United States).

### 2.7 Jejunal mRNA transcriptome sequencing and Q-PCR validation

On 28th day, lamb jejunum (2 cm) was collected into a tube containing Trizol, and the samples were put in the dry ice and sent to the laboratory. Total RNA was extracted by the Trizol reagent method, and the RNA concentration was detected by nanodrop method. All the RNA concentrations were greater than 30 ng/μL, the 260/280 ratio was between 1.8 and 2.2, and the extracted RNA was detected. It satisfies the detection requirements. Most of the total RNA was sent to Shanghai Majorbio Co., Ltd. (Shanghai, China) for mRNA transcriptome sequencing, and the remaining samples were subjected to cDNA for Q-PCR conformation of key pathway genes was replicated (Qi et al., 2024).

According to the operation instructions of the SYBR (R) Premix Ex Taq TMⅡ fluorescence quantitative PCR kit, the mRNA expression levels of key genes CD40, MHC II, CCL28, TCFβ1, Integrinα4, BAFFR, pIgR, CD19, CD72, Igα (CD79α), and Igβ (CD79b) were determined by Q-PCR method. The sheep lamb’s mRNA sequences were attained from NCBI, and primer was conducted using Primer 5 software as shown in Table 2.

**TABLE 2:** Primer’s sequence of qPCR.

Gene	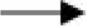 Primer sequence (5′-3′)	Base number
CD40	GTA​CCA​GTC​ACA​CCT​GCG​AA	20
	TGA​TAC​AGG​CAG​ATA​CCA​ACA​GG	23
MHC Ⅱ	ACC​CCT​ACA​TCA​GAG​CTG​ACA	21
	AAT​GAA​GAC​TTT​CCT​CTC​CTT​CC	23
CCL28	GAC​GTG​TCG​CAT​CCA​GAG​AG	20
	ACA​CAG​ACT​CTT​CTG​CGC​TT	20
TGFβ1	CTG​GAC​ACG​CAG​TAC​AGC​AA	20
	ACA​GGA​AAG​ACT​TGT​GTC​GC	20
Integrin α4	CCA​ACA​AAC​ACA​TAT​TCA​TGG​CAG	24
	GAG​TGG​CCT​GCT​AGA​TGT​GA	20
BAFFR	CCA​TGT​CTT​TGG​GGA​CGA​ACT​G	22
	TGC​CTG​GAA​GGG​AAT​GAT​TGG	21
pIgR	ATC​AAC​TGC​CCC​TTC​ACG​AG	20
	GGT​ATA​GCT​GCC​GCT​CAC​AT	20
CD19	TAG​TGG​AGG​CTA​AAG​AGG​GAG​G	22
	CCA​GGC​CAG​TTG​TTC​AGG​G	19
CD72	GTG​CTC​CTC​CCC​AGA​TTC​CT	20
	ATG​GAT​GAG​AAC​TGT​TGC​TGG​T	22
Lgα (CD79α)	CGG​AAA​CGA​TGG​CAG​AAC​AT	20
	TCT​AGG​TTC​AGG​CCC​TCA​TAG​A	22
Lgβ (CD79b)	CCA​GGC​AGT​TAC​ACG​TTT​TCC	21
	CCG​GGA​ACA​AGT​GTT​TCC​TTT	21
β-actin	GTC​ACC​AAC​TGG​GAC​GAC​AT	20
	AGG​TCT​CAA​ACA​TGA​TCT​GGG​T	22

### 2.8 Statistics analysis

Experimental data were analyzed using IBM SPSS Statistics 24 software by one-way ANOVA-Duncan multiple comparisons and reported the results as “X ± SE.” The statistical significance was determined at the *p* < 0.05 level.

## 3 Results and analysis

### 3.1 Changes of polysaccharide content in *Alhagi maurorum* Medik at different growth stages in different regions

As shown in Table 3, with the passage of the growth period, the polysaccharide content of *A. maurorum* Medik produced in Aksu area and Shanshan area first showed an increase and then a decrease trend, and the polysaccharide content reached the highest level at the maturity stage. The polysaccharide content of SS was significantly (*p* < 0.05) higher than AK at the flowering stage, maturity stage, and withered stage.

**TABLE 3:** Dynamic change of AP content in different regions (%).

Sampling point	Vegetative period	Flowering stage	Maturity stage	Withered stage
AK	4.07 ± 0.04^b^	7.75 ± 0.37^b^	11.28 ± 0.62^b^	9.52 ± 0.24^a^
SS	4.41 ± 0.05^a^	8.69 ± 0.14^a^	12.37 ± 0.09^a^	7.86 ± 0.15^b^

Note: Different lowercase letters indicate significant differences (*p* < 0.05), the same or no letter indicates no significant difference (*p* > 0.05), the same below.

### 3.2 Effects of SS and AK on organ index of lambs

**FIGURE 2:**
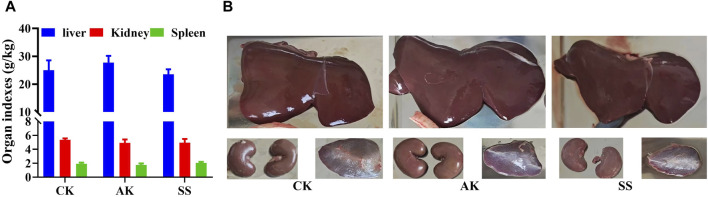
Effects of SS and AK on organ index of lambs. **(A)** Organ index, **(B)** picture of sheep lamb’s organs.

Figure 2 showed that there was non-significant difference in liver index, kidney index and spleen index among CK, AK, and SS groups (*p* > 0.05). Our results indicate that AP in different regions has non-significant role in the development of immune organs of lambs.

### 3.3 Effects of SS and AK on the immunoglobulin content of lambs

**FIGURE 3:**
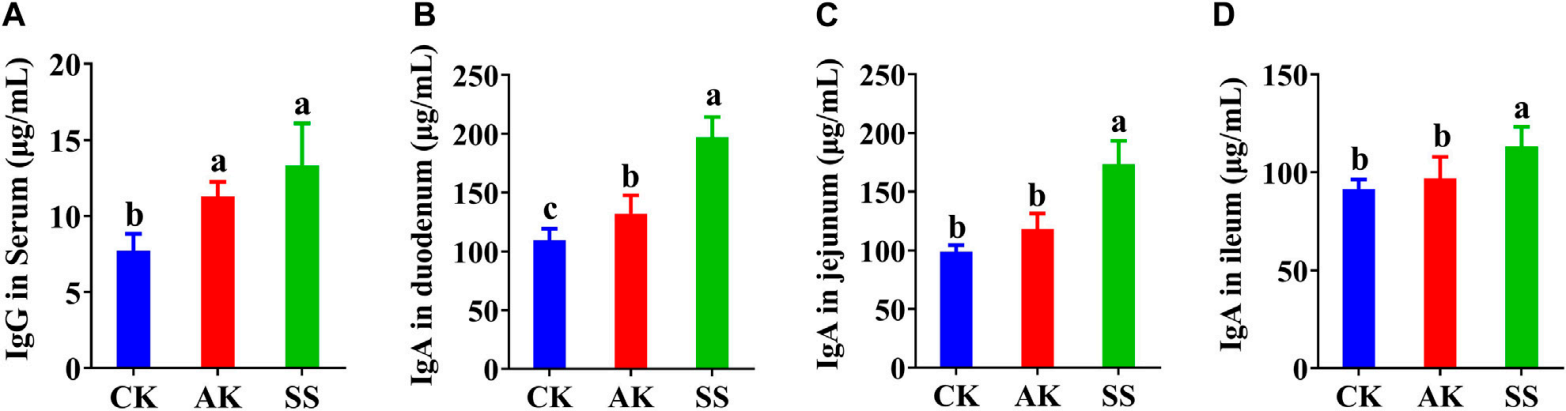
Effects of SS and AK on antibody levels of lambs. **(A)** serum IgG, **(B)** duodenum IgA, **(C)** jejunum IgA, and **(D)** ileum IgA. Note: Different lowercase letters indicate significant differences (*p* < 0.05), the same applies below.

Serum lgG and lgA contents were detected in different parts of intestine, respectively. As shown in Figure 3A, serum IgG in the AK and SS groups was significantly (*p* < 0.05) increased in contrast to CK group. As shown in Figures 3B–D compared with CK and AK groups, the contents of IgA in the duodenum, jejunum, and ileum in the SS group were significantly higher (*p* < 0.05).

### 3.4 Effects of SS and AK on serum cytokine levels in lambs

**FIGURE 4:**
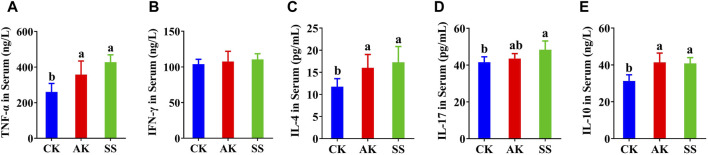
Effects of SS and AK on serum cytokine content of lambs. **(A)** serum TNF-α, **(B)** serum INF-γ, **(C)** serum IL-4, **(D)** serum IL-17, **(E)** serum IL-10.

Effects of AP on Th0, Th1, Th2, Th17, and Treg-associated cytokines TNF-α, INF-γ, IL-4, IL-17, and IL-10 were detected. As shown in Figures 4A, C, E the contents of TNF-α, IL-4, and IL-10 in AK and SS groups were significantly enhanced than those in CK group (*p* < 0.05), while the INF-γ (Figure 4B) in CK group was no difference in AK and SS groups (*p* > 0.05). As shown in Figure 4D, the content of IL-17 in the SS group was meaningfully higher (*p* < 0.05) than that of the CK group, while no prominent difference was noticed in the CK and AK groups (*p* > 0.05).

### 3.5 Effects of SS and AK on intestinal cytokine levels in lambs

**FIGURE 5:**
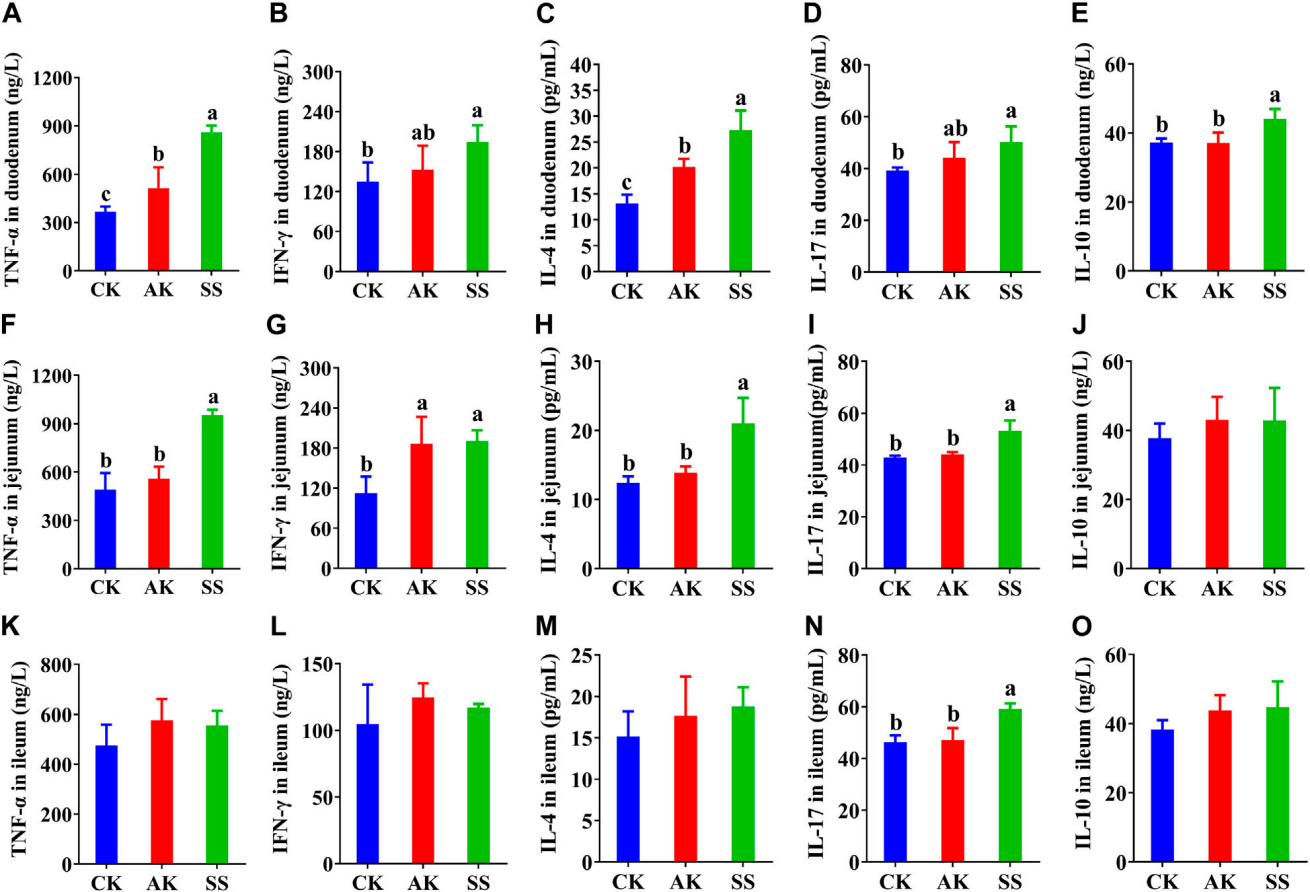
Effects of SS and AK on intestinal cytokine content of lambs. **(A–E)** duodenum cytokine content, **(F–J)** jejunum cytokine content, **(K–O)** ileum cytokine content.

The contents of cytokines were detected in duodenum, jejunum, and ileum of lambs, respectively. The contents of TNF-α, INF-γ, IL-4, IL-17 and IL-10 in the duodenum (Figures 5A–E), the contents of TNF-α, INF-γ, IL-4, and IL-17 in the jejunum (Figures 5F–I), and the contents of IL-17 in the ileum (Figure 5N) in the SS group were significantly (*p* < 0.05) increased than those in CK group. Whereas, the contents of TNF-α, IL-4, and IL-10 in the duodenum (Figures 5A–E), TNF-α, IL-4, and IL-17 in the jejunum (Figures 5F, H, I), and IL-17 (Figure 5N) in the ileum in SS group were more significantly (*p* < 0.05) than AK and CK groups.

### 3.6 Effects of SS and AK on intestinal and spleen morphology of lambs

**FIGURE 6:**
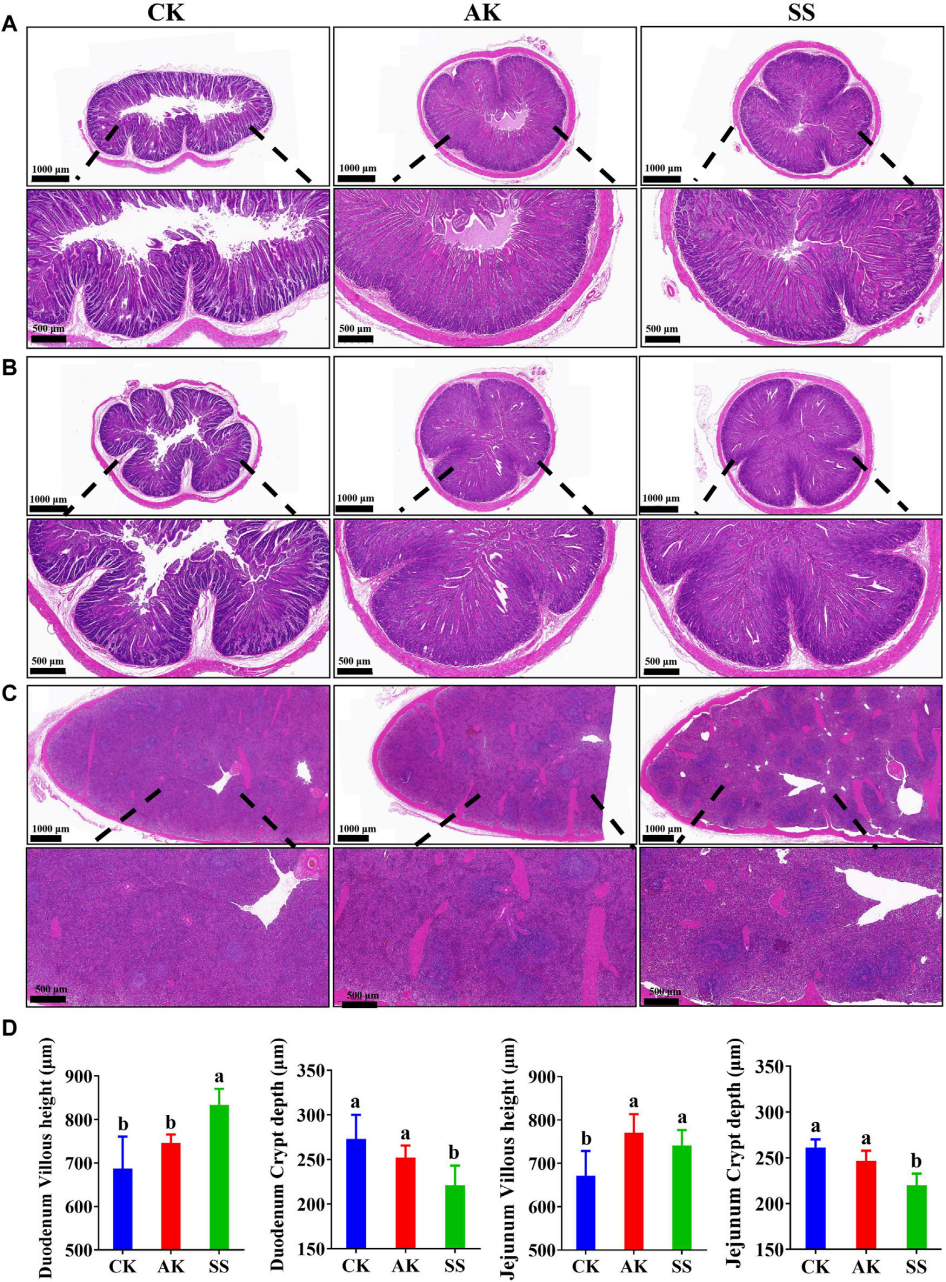
Effects of SS and AK on intestinal and spleen morphology of sheep lambs. **(A)** HE stained section of duodenum, **(B)** HE stained section of duodenum jejunum, **(C)** HE stained section of spleen, **(D)** Villus length and crypt depth of duodenum and jejunum.

The results of H&E-stained sections of different segments of small intestine and spleen of lambs are as follows. As shown in Figures 6A–C, there is no abnormality in the pathological structures of the duodenum, jejunum, and spleen in all groups. The intestinal villi of the duodenum and jejunum were organized in all groups, and there were no inflammatory cell infiltrates or other lesions in intestinal and spleen cells. As shown in Figures 6A,D, the duodenal villus height in the SS group was significantly higher than the CK and AK groups (*p* < 0.05), and the crypt depth was significantly lower than CK and AK groups (*p* < 0.05). The villus height of the jejunum in the SS and AK groups was increased significantly (*p* < 0.05) than CK group, and the crypt depth in the SS group was decreased significantly (*p* < 0.05) than CK and AK groups. Compared with the CK group, the volume of spleen corpuscles in the SS and AK groups increased, and the boundary between the red pulp and the white pulp was cleared.

### 3.7 Results of general intestinal gene change and KEGG enrichment analysis in lambs

**FIGURE 7:**
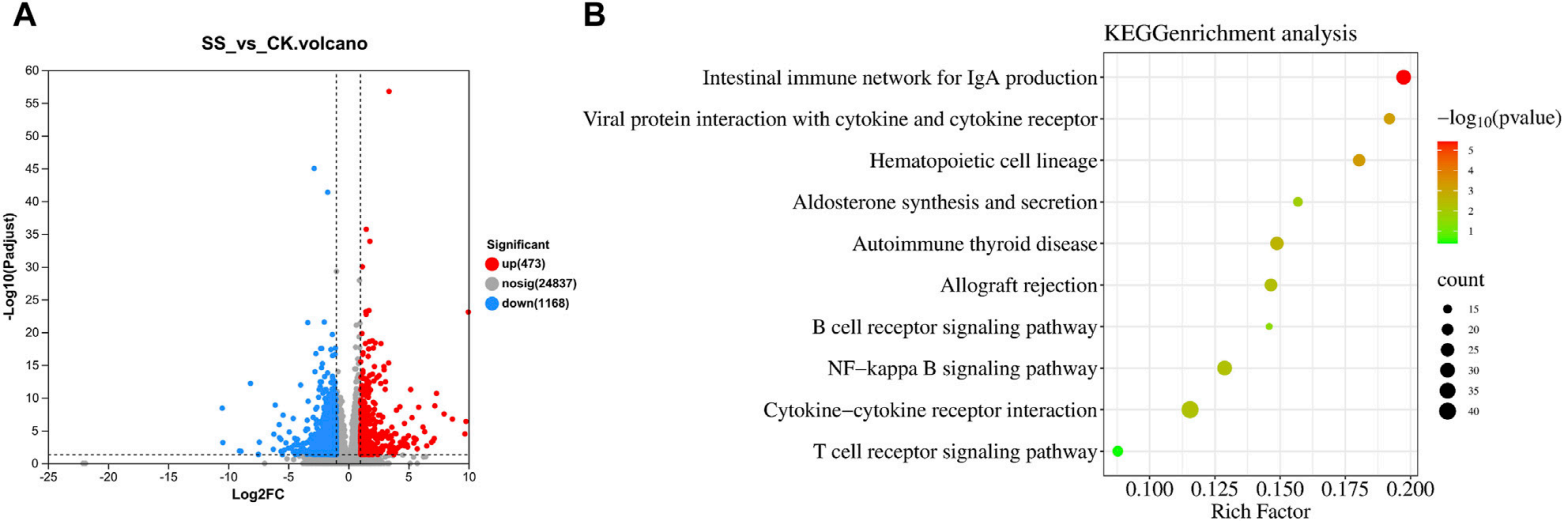
**(A)** The change of gene expression and **(B)** KEGG enrichment analysis.

As shown in Figure 7A, there was 1641 different genes in SS and CK groups, while significantly upregulated 473 genes and downregulated 1168 genes in SS group compared with CK group. The KEGG enrichment shown in Figure 7B, the SS group activated and regulated intestinal immune function through 10 pathways, such as intestinal immune network for IgA production pathway (*p* < 0.001), interaction of cytokines and cytokine receptors with viral protein (*p* < 0.001), hematopoietic cell lineage (*p* < 0.001), aldosterone synthesis and secretion (*p* < 0.05), autoimmune thyroid disease (*p* < 0.01), allograft rejection (*p* < 0.01), B cell receptor signaling pathway (*p* < 0.05), NF-kappa B signaling pathway (*p* < 0.01), cytokine-cytokine receptor interaction (*p* < 0.01), and T cell receptor signaling pathway (*p* = 0.4016).

### 3.8 Differential gene expression cluster analysis and related pathways in lamb intestinal

**FIGURE 8:**
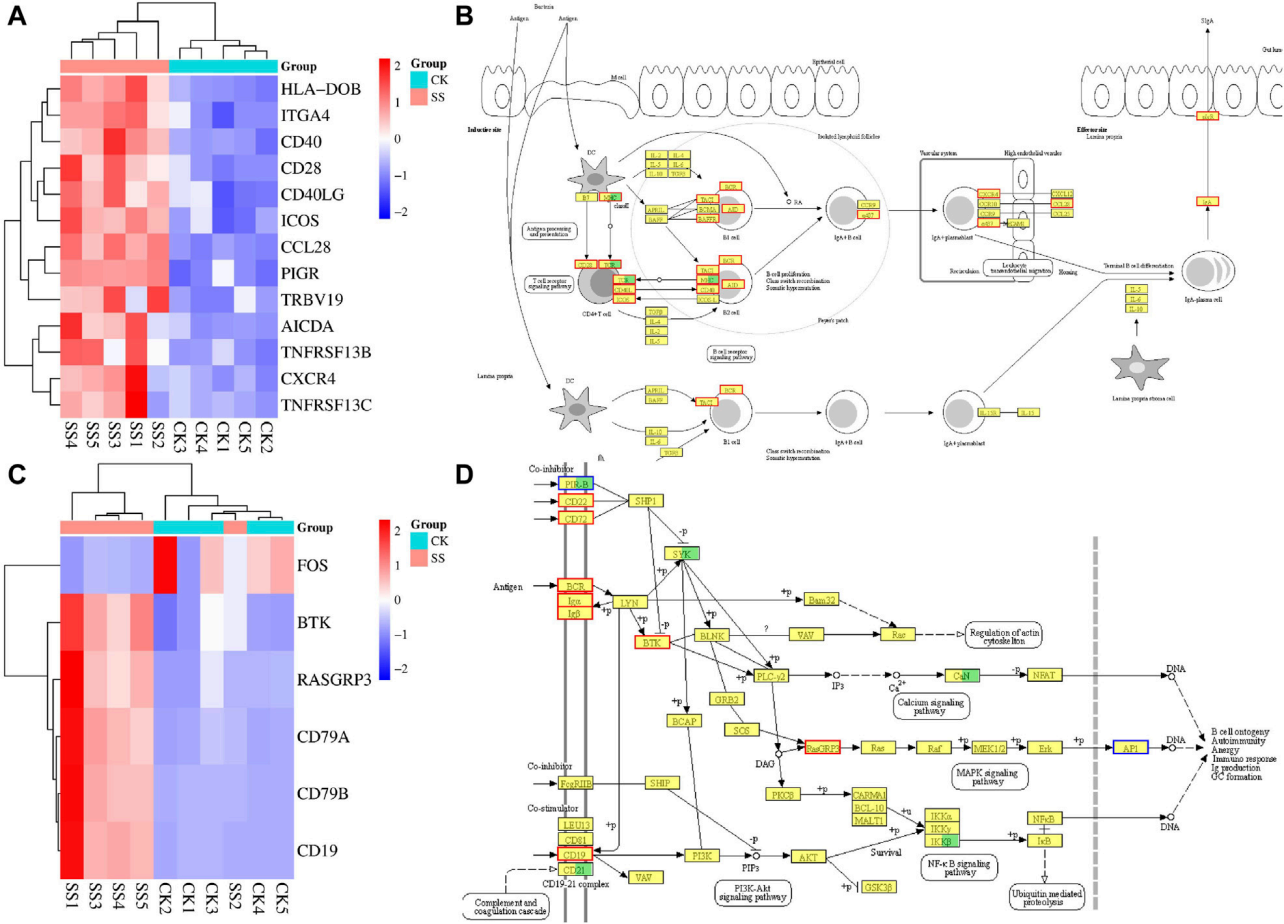
The gene expression cluster analysis and pathway simulation map. **(A)** gene expression cluster analysis of intestinal immune network for IgA production pathway, **(B)** simulation map of intestinal immune network for IgA production pathway, **(C)** gene expression cluster analysis of B cell receptor signaling pathway, **(D)** simulation map of B cell receptor signaling pathway.

KEGG enrichment analysis showed that the SS group enhance intestinal immune function of sheep lambs through IgA production pathway. Therefore, gene expression cluster analysis and pathway simulation analysis were performed for differential expressed genes in the intestinal immune network related to IgA production pathway and B cell receptor signaling pathway related to IgA production. As shown in Figures 8A,B, in the IgA production pathway and pathway simulation map, the SS group significantly upregulated 13 genes and 14 genes compared with the CK group, respectively. The functional genes co-up-regulated in both pathways were CD40, CD28, ICOS, CCL28, and PIGR. The Figures 8C,D showed that in the B cell receptor signaling pathway and pathway simulation map, the SS group significantly upregulated 5 genes and 8 genes compared with the CK group, respectively. The functional genes co-up-regulated in both pathways were BTK, RASGRP3, and CD19.

### 3.9 Q-PCR detection results of key genes in the pathway

**FIGURE 9:**
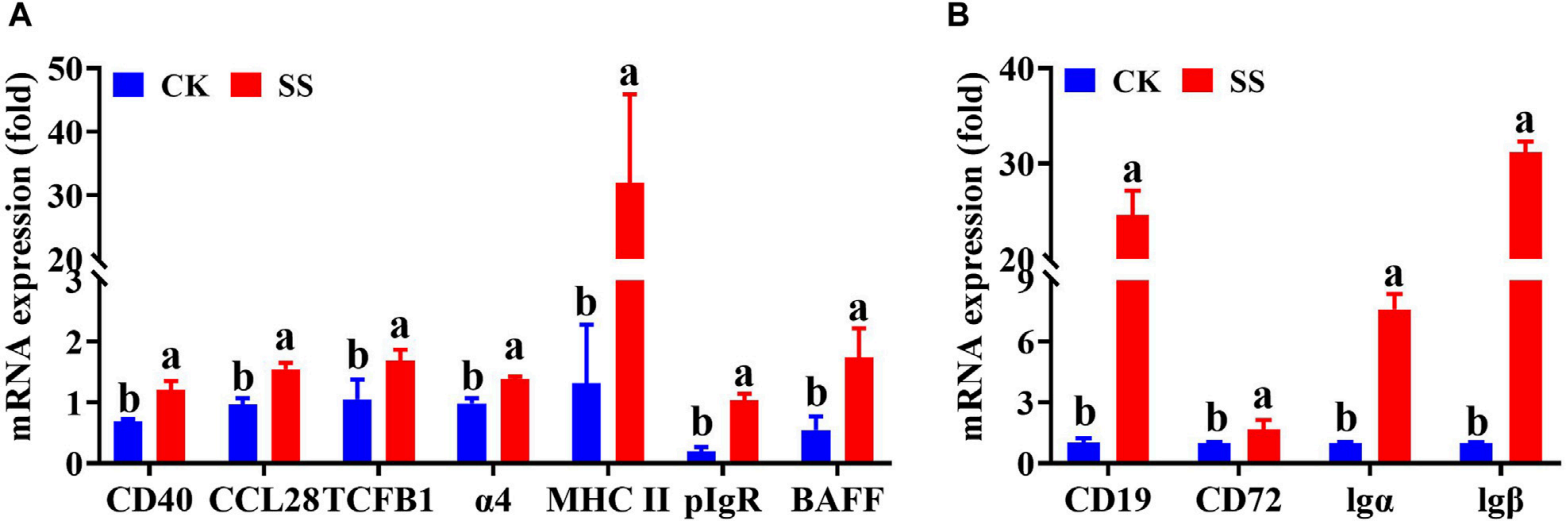
mRNA expression level of key in the Intestinal immune network for IgA production pathwayand B cell receptor signaling pathway. **(A)** detection of key genes in intestinal immune network for IgA production pathway, **(B)** detection of key genes in B cell receptor signaling pathway.

The key regulatory genes in the above differential gene expression cluster analysis and corresponding pathway maps were confirmed by q-PCR. As shown in Figure 9A, the expressions of CD40, MHC II, CCL28, TCFβ1, BAFFR, pIgR, and Integrin α4 genes in the SS group were significantly increased (*p* < 0.05) than CK group. Among them, BAFFR, pIgR, CD40, CCL28 and Integrin α4 genes were significantly enhanced by differential gene expression cluster analysis, pathway simulation map, and q-PCR validation of the intestinal immune network for IgA production pathway. As shown in Figure 9B, the expressions of CD19, CD72, Igα (CD79α) and Igβ (CD79b), genes in the SS group were significantly higher than those in the CK group (*p* < 0.05). Among them, differential gene expression cluster analysis, pathway simulation map, and q-PCR validation of the B cell receptor signaling pathway have significantly enhanced in Igα、lgβand CD19 genes.

## 4 Discussion

The intestine is the largest part of digestive system and immune organ in the animal body. The intestine is barrier in preventing the invasion of pathogens and maintaining the homeostasis of the host environment (Hachimura et al., 2018). The IgA is secreted into the intestinal cavity to form protective layer in the intestinal mucosa to neutralize invading pathogens for damaging the intestinal tissue (Pietrzak et al., 2020). Serum IgG plays a significant role in humoral immunity and inhibits the invasion of pathogens to protect the intestine from infection through regulating the mucosal immune system (Roomruangwong et al., 2017). The effect of SS and AK on the intestinal immune function of lambs is shown in Figure 3. The serum IgG content in the AK and SS groups were significantly higher as compared to CK group, and IgA content in the duodenum, jejunum, and ileum in the SS group were significantly (*p* < 0.05) higher than AK and CK groups. The results indicated that SS has better effect on enhancing intestinal immunity, and SS enhanced the immune function by increasing the immunoglobulin in serum and intestinal tissue.

Cytokine is a significant parameter to evaluate the immune capacity of the body and plays an important role in the activation, proliferation, and differentiation of dendritic cells (DCs), T cells, and B cells, which are key factors in regulating serum IgG and intestinal IgA secretion (Wang et al., 2021). The Th0-associated cytokines TNF-α and Th1-related cytokines INF-γ can promote the activation and proliferation of DCs, T cells and B cells. Thereby it is protecting the mucosal barrier integrity and enhancing intestinal immune responses (Pesce et al., 2022). The Th2-related cytokines IL-4 can promote the differentiation of B cells in intestinal Peyer’s patch lymph nodes to produce IgA antibodies, and also the differentiation of B cells to produce IgG antibodies (Zhao et al., 2022). The interplay of Th17-associated cytokines IL-17 and Treg-associated cytokines IL-10 helps to maintain the homeostasis of intestinal immunity (Sawant et al., 2019; Gribonika et al., 2022). The cytokines IL-17, and IL-10 can promote the proliferation of intestinal epithelial cells, production of mucus and antimicrobial peptides in intestinal epithelial cells, enhance the integrity of intestinal mucosal barrier, and prevent the pathogen invasion and infection (Gribonika et al., 2022). Our results indicated that both SS and AK have good intestinal immuno-enhancing effects, while the intestinal immune-enhancing activity of SS group is stronger than CK. SS promotes the secretion of TNF-α, IL-4, IL-17, and IL-10 in the intestine and serum (Figure 4; Figure 5), increases the activation and proliferation of intestinal DCs, T cells, and B cells, and increases the IgA antibody content in the intestinal mucosal layer, thereby increasing the intestinal mucosal barrier and intestinal immune function.

Intestinal function can be reflected by the morphology of the intestine. Intestinal villi greatly reflect the ability of the intestine to absorb nutrients, and intestinal villi cover the intestinal mucosal layer to prevent the invasion of pathogens. The depth of intestinal crypts represents the rate of cell generation (Zhu et al., 2022). The smaller the crypts, the better the cell maturation, the higher the efficiency of nutrient absorption in the intestine. Meanwhile, the crypts containing a large number of immune cells will beneficial for the immune cells to regulate the intestinal immune response (Xiong et al., 2015). The newborn lambs were fed with bovine colostrum and found that the intestinal villi height increased significantly and crypt depth decreased after feeding bovine colostrum, indicating that bovine colostrum has beneficial effect to promote intestinal growth and maturation in lambs (Zhu et al., 2022). Lambs with early weaning stress can suffer into intestinal morphological damage. Furthermore, compared with normal weaned lambs, early weaned lambs have significantly decreased intestina lgA antibody content, shortened jejuna villi and increased crypt depth. But after yeast peptide treatment, the length of jejuna villi increased and crypt depth was decreased, which showed its good therapeutic effect on intestinal injury and intestinal immune recovery (Li et al., 2023). Cai et al. treated cyclophosphamide immunosuppressant model mice with *A. maurorum* Medik Fruit polysaccharide (AH) and found that AH could significantly increase the height of villi and reduce the crypt depth of duodenum, jejunum and ileum of mice, which is good for immune system (Cai et al., 2021a). Our results showed that the villus height of the duodenum in the SS group was significantly higher than that of CK and AK groups (*p* < 0.05), and the crypt depth of the duodenum and jejunum in the SS group was significantly (*p* < 0.05) lower than that in CK and AK groups (Figure 6). The results showed that SS was more potent for promoting intestinal growth and maturation, intestinal nutrient absorption capacity, and enhancing intestinal barrier function and immune function in lambs. The overall immunity of an animal may be reflected to some extent by the immune status of the spleen (Aliyu et al., 2021). Both the spleen and the intestine contain a large number of immune cells, which communicate with each other through the lymphatic circulation to jointly deal with external pathogens. So, the immune function of the spleen is stronger than the immune function of intestine (Bronte and Pittet, 2013; Aliyu et al., 2021). The results of this study showed that, compared with the CK group, the number and volume of spleen cells in the AK and SS groups were increased, and the boundary between the red pulp and white pulp was prominent (Figure 6C), which indicates that the AK and SS could effectively enhance the immune function of the spleen in lambs. Compared with AK group, SS can effectively promote the expression of intestinal IgA and various cytokines, and promote intestinal development and immune function. The difference in activity between SS and AK may be due to the difference in composition or structure of *A. maurorum* Medik polysaccharide produced in different regions.

The intestinal immune network for the IgA production is the most important pathway regulating the production of intestinal IgA antibodies (Doron et al., 2023). This pathway mainly regulates intestinal IgA production by activating one or both of B cell activation pathways with T cell-independent pathways or T cell-dependent pathways (Conrey et al., 2023). The intestinal IgA antibodies are secreted by activated B cells; thus, IgA secretion is influenced by B cell activation and B cell number (Biswas et al., 2021). Our results showed (Figure 7B) that compared with the CK group, the SS group significantly upregulated the intestinal immune network for IgA production pathway (*p* < 0.001) and B cell receptor signaling pathway (*p* < 0.05), which indicates that SS has a beneficial effect of activation on both pathways regulating IgA secretion. Further exploration of the expression of key genes in the above pathways showed that, compared to CK, BAFFR, pIgR, CD40, CCL28, Integrin α4, Igα, Igβ and CD19 genes were significantly higher in the SS group (Figures 8, 9). It is known that BAFFR is a key gene in the T cell-independent pathway, and CD40, CCL28, and Integrin α4 are key genes in the T cell-dependent pathway. The pIgR is a key gene that regulates IgA to become secretory sIgA, so that IgA can be effectively secreted into the intestinal mucosa. Igα, Igβ, and CD19 are key genes that regulate the development, activation, and proliferation of B cells. According to the results of KEGG enrichment, pathway simulation, and Q-PCR, SS activated T cell-independent pathways, T cell-dependent pathways, and B cell proliferation and activation of lgA in lamb’s intestine. Which secrete a large amount of IgA in the intestinal mucosal layer by highly expressing of pIgR.

## 5 Conclusion

In conclusion, SS can effectively increase the serum IgG, IgA, and cytokine contents of the duodenum, jejunum, and ileum, which improves the villus height and crypt depth of the duodenum and jejunum, and increase the spleen volume. SS can induce intestinal secretion of IgA by activating T cell-dependent pathways, T cell-independent pathways, and activation and proliferation of B-cells to protect the intestine. The difference in the activity of SS and AK may be due to the difference in polysaccharide composition. In conclusion, among the polysaccharide produced in the two regions, SS can be used as a natural feed with good immune-enhancing effects in the intestines of lambs.

## Data Availability

The original contributions presented in the study are included in the article/Supplementary Material, further inquiries can be directed to the corresponding authors.
