# Supplementary material for: Effects of Alhagi maurorum Medik polysaccharide derived from different regions on the intestinal immune functions of lambs
Source: Front Pharmacol. 2024 Jul 15;15:1422461. doi: 10.3389/fphar.2024.1422461 (PMC11284127; doi:10.3389/fphar.2024.1422461)
Supplement: Supplementary file 10 [file DataSheet1.docx]

Chromatographic condition

Chromatographic column: Agilent Eclipse XDB C18 (4.6 x 250 mm, 5 µm)

Gradient condition

A: Acetonitrile; B: 0.1% formic acid

| Time | A（%） | B（%） |
| --- | --- | --- |
| 0 | 5 | 95 |
| 15 | 20 | 80 |
| 25 | 25 | 75 |
| 35 | 100 | 0 |

Processing software: TCM Chromatographic Fingerprint Similarity Evaluation System (2012.130723 edition)

Similarity calculation results

|  | S1 | S2 | Control fingerprint |
| --- | --- | --- | --- |
| S1 | 1.000 | 0.980 | 0.998 |
| S2 | 0.980 | 1.000 | 0.991 |
| Control fingerprint | 0.998 | 0.991 | 1.000 |

Note: S1(*Alhagi maurorum* Medik from Shanshan ), S2(*Alhagi maurorum* Medik from Aksu )


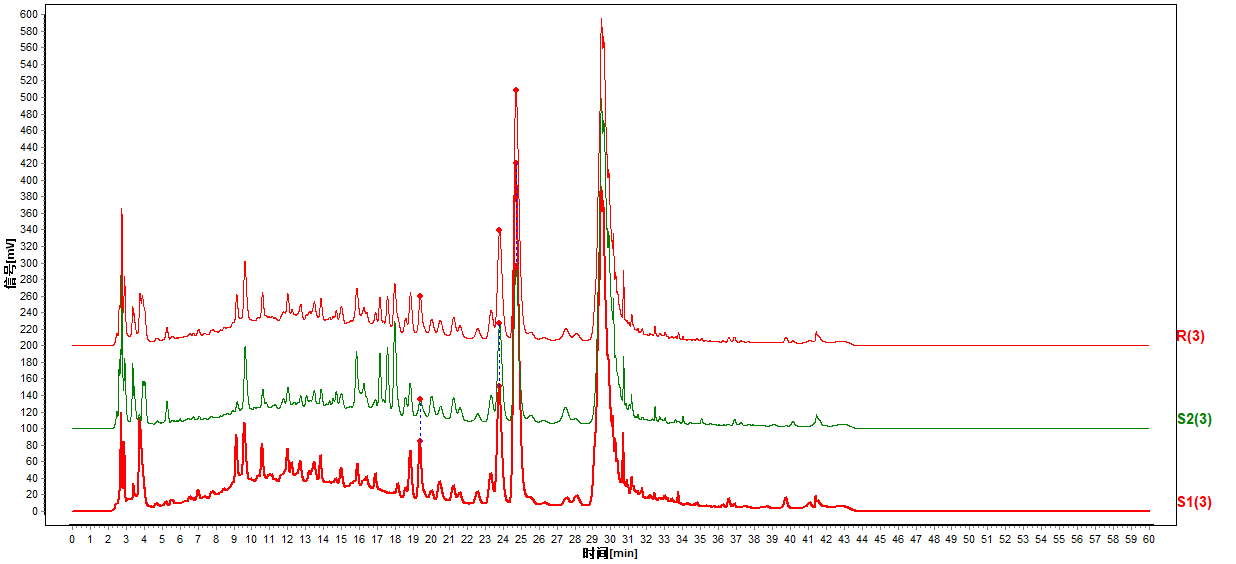


Fingerprint map identification

t1:9.193min, t2:9.583min, t3:10.642min, t4:12.035min, t5:15.823min, t6:17.105min, t7:17.606min, t8:17.996min, t9:18.888min, t10:19.389min, t11:23.791min, t12:24.738min

Common peak

| Common peak serial number | Retention time (min) |
| --- | --- |
| 1 | 9.193 |
| 2 | 9.583 |
| 3 | 10.642 |
| 4 | 12.035 |
| 5 | 15.823 |
| 6 | 17.105 |
| 7 | 17.606 |
| 8 | 17.996 |
| 9 | 18.888 |
| 10 | 19.389 |
| 11 | 23.791 |
| 12 | 24.738 |

According to the HPLC superposition spectra of S1(*Alhagi maurorum* Medik from Shanshan ) and S2(*Alhagi maurorum* Medik from Aksu ), the two batches of*Alhagi maurorum* Medik were similar. Through the similarity evaluation system of TCM chromatographic fingerprint (2012.130723 edition) with a time width of 0.1, automatic matching was adopted to generate control map R, and a total of 12 peaks were determined. Through the similarity calculation, it can be concluded that the two batches of *Alhagi maurorum* Medik have high similarity. However, there were differences in the common peak height of the two batches of *Alhagi maurorum* Medik, which indicated that the content of main components of *Alhagi maurorum* Medik was different in different places, which may be related to local climate conditions.
